# Vitamin A Inhibits Development of Dextran Sulfate Sodium-Induced Colitis and Colon Cancer in a Mouse Model

**DOI:** 10.1155/2016/4874809

**Published:** 2016-05-19

**Authors:** Isao Okayasu, Kiyomi Hana, Noriko Nemoto, Tsutomu Yoshida, Makoto Saegusa, Aya Yokota-Nakatsuma, Si-Young Song, Makoto Iwata

**Affiliations:** ^1^Department of Pathology, School of Medicine, Kitasato University, Sagamihara, Kanagawa 252-0374, Japan; ^2^Research Center for Biological Imaging, School of Medicine, Kitasato University, Sagamihara, Kanagawa 252-0374, Japan; ^3^Laboratory of Immunology, Kagawa School of Pharmaceutical Sciences, Tokushima Bunri University, Sanuki, Kagawa 769-2193, Japan; ^4^Institute of Neuroscience, Kagawa School of Pharmaceutical Sciences, Tokushima Bunri University, Sanuki, Kagawa 769-2193, Japan

## Abstract

Vitamin A is essential to mucosal immunity and cell differentiation. The fact that lack of it might involve chronic inflammation and increased risk of cancer has been reported. Little is known about the mechanism of vitamin A deficiency in the development of colitis and its influence on development of colorectal cancer. To determine the influence of vitamin A deficiency on colitis and colorectal cancer development, an experimental study using a colitis mouse model was performed. Dextran sulfate sodium (DSS) colitis was induced in vitamin A-deficient and vitamin A-supplemented mice. Further, colorectal carcinoma was induced by a combination of azoxymethane preinjection and DSS colitis. Results were compared between the two groups mainly by immunohistochemical analysis. Colitis was more severe and recovery from colitis was slower in vitamin A-deficient mice than in vitamin A-supplemented mice. Compared with vitamin A-supplemented mice, vitamin A-deficient mice had decreases in colonic subepithelial myofibroblasts and the ratio of mucosal IgA^+^/IgG^+^ cells, increases in CD11c^+^ dendritic cells, and a higher rate of development of colorectal carcinoma with colitis following azoxymethane. Vitamin A lipid droplets in subepithelial myofibroblasts were decreased in vitamin A-deficient mice, suggesting alterations in colonic crypt niche function. Thus, vitamin A inhibited colitis and the development of colorectal cancer.

## 1. Introduction

The influence of vitamin A and its deficiency on mucosal immunity and cell differentiation has been widely explored and lack of it has been reported to involve chronic inflammation and increased risk of cancer. Through a retrospective chart review it was revealed that deficiencies of vitamins D and A and zinc were relatively common in children and young adults with newly developed inflammatory bowel diseases [1]. Also, low levels of serum vitamins A and E were shown in children and young adults with active inflammatory bowel disease [2].

Further, vitamin A deficiency enhanced the T-helper type 1 (Th1) response and elevated levels of proinflammatory cytokines in obese individuals, suggesting an increased inflammatory response [3]. In rats with vitamin A deficiency downregulation of Retinoic Acid Receptor- (RAR-) *α* mRNA, increased dendritic cells, and increased protein secretion of IL-12 in the intestinal mucosa were shown. Inversely, supplemental vitamin A in an in vitro culture system of Peyer's patches promoted maturation of dendritic cells and upregulation of RAR-*α* mRNA, suggesting the possibility of a reduction in intestinal inflammation [4]. Kang et al. reported that a high vitamin A level induced a CCR9^+^ *α*4*β*7^+^ FoxP3^+^ T cell subset, suppressive of intestinal inflammation in SAMP1/YP mice, which had the characteristic of developing spontaneous transmural ileitis like Crohn's disease [5]. These findings suggest that vitamin A is important in maintaining appropriate mucosal immunity and in regulating intestinal inflammation. Considering the above findings, there may be an association of vitamin A levels with intestinal inflammation.

Retinoids, vitamin A metabolites, are essential for epithelial differentiation and control of epithelial and mesenchymal interaction through* Ret* expression [6]. Concerning carcinogenesis, retinoids are effective in chemoprevention and differentiation therapy for cancers of various organs [7]. Further, an association of vitamin A deficiency with cervical intraepithelial neoplasia in HIV-infected women was reported [8] as was chemically induced nephroblastoma in rats [9]. CYP26A1, the gene encoding the cytochrome P450 enzyme which is a major retinoic acid-catabolic enzyme, was highly expressed in cancers of various organs [10–13]. Decreased intracellular retinoic acid or a deficiency due to high CYP26A1 expression induced cells into highly proliferative and invasive states [13] and promoted significant resistance to apoptosis, possibly contributing to the carcinogenic process [14]. These findings may indicate that vitamin A deficiency promotes carcinogenesis [15]. Regarding nutrition, inverse associations were shown between the risk of colon cancer and the use of multivitamin and calcium supplements, although direct associations between colon cancer risk and supplemental vitamin A and vitamin C were inconsistent [16].

Previously we revealed that subepithelial myofibroblasts corresponded to colonic stellate cells containing vitamin A lipid droplets, which are thought to play an important role in the maintenance of the niche function of colonic stem cells [17–20]. For this report, we examined whether vitamin A inhibits intestinal inflammation and the development of inflammation-associated colon cancer using our previously developed DSS colitis mouse model [21, 22].

## 2. Materials and Methods

### 2.1. Mice

BALB/c mice were purchased from CLEA Japan (Tokyo, Japan) and maintained under specific pathogen-free conditions in our animal center. Vitamin A-deficient and vitamin A-supplemented BALB/c female mice were produced according to a previously described method [23, 24]. Briefly, mice were bred, and gravid females received either a chemically defined diet that lacked vitamin A (modified AIN-93M feed, Oriental Yeast, Tokyo, Japan) or a vitamin A-supplemented control diet containing retinyl acetate (5,000 IU/kg in the modified AIN-93M feed). This concentration is appropriate as a supplement considering the natural oxidative degradation of vitamin A [24, 25]. These diets were started before gestation. The mouse pups were weaned at 4 weeks of age and maintained on the same diet until the experiment finished.

### 2.2. Induction and Assessment of Colitis

Colitis was induced with administration of synthetic dextran sulfate sodium (DSS, mol wt 54,000, Ensuikou Sugar Refining Co., Ltd., Chuo-ku, Tokyo, Japan), a procedure that we originally developed [21]. Briefly, mice were divided into two groups, vitamin A-deficient and vitamin A-supplemented groups, and given distilled drinking water containing 0% or 1% (wt/vol) DSS ad libitum under the regimen established for the experiment. After drinking 0% or 1% DSS for 7 or 12 days, mice were killed. The removed colon was put on thick qualitative filter paper, exposed inside out by cutting longitudinally, and fixed in 10% formalin solution. Longitude of each colon was measured. Histological examinations were performed with H&E staining of paraffin sections of the longitudinal sections of the colons [21]. Total colon was equally divided into three segments, the proximal, middle, and distal segments, to assess the distribution of colitis. Severity of colitis at each part was graded on a scale from 0 to 3 and expressed as the pathological index according to the standard scoring system: 0, normal; 1, focal inflammatory cell infiltration including polymorphonuclear leukocytes; 2, inflammatory cell infiltration with gland dropout and/or crypt abscess; and 3, mucosal ulceration. The sum of each colitis score at three segments was shown as total colitis score.

### 2.3. Induction of Colonic Tumors

For induction of colonic tumors, mice were administered an intraperitoneal injection of azoxymethane (7.4 mg/kg, Sigma-Aldrich Corp., St. Louis, MO, USA), which was followed beginning two weeks later by administration of 1% DSS for 7 days. After drinking distilled water for 3 weeks, the mice were killed. All polypoid or flat elevated lesions that developed were histopathologically counted by observation of a longitudinal paraffin section with H&E staining [22].

### 2.4. Electron Microscopic Examination

For electron microscopic observation, the colonic wall was fixed by 2.5% (vol/vol) glutaraldehyde in a solution of phosphate buffer (pH 7.2) and by 2% (wt/vol) osmium tetroxide in a similar phosphate buffer solution (pH 7.2). After Epon embedding, ultrathin sections were stained with uranyl acetate and lead citrate followed by electron microscopic examination.

### 2.5. Immunohistochemical Analysis

Immunohistochemical staining was performed as shown in Table 1. Frozen sections of colonic mucosa after 2% paraformaldehyde fixation were supplied for examination of CD11c^+^ dendritic cells. Formalin-fixed paraffin sections were used for analysis of *α*-SMA^+^, IgM^+^, IgG^+^, and IgA^+^ cells. Sections were incubated with the primary antibodies at the given dilution overnight at 4°C. 3,3′-Diaminobenzidine was applied as the final chromogen, and nuclei were counterstained with methyl green solution to facilitate histopathological assessment.

### 2.6. Statistical Analysis

Results are summarized as means ± standard deviation (SD). Data were statistically analyzed by the Mann-Whitney *U* test and Chi-squared test using Stat View ver. 5.0 for Windows (SAS Institute Inc., Cary, NC, USA). All* p* values < 0.05 were considered statistically significant.

## 3. Results

### 3.1. Modifications of Intestinal Homeostasis in Vitamin A-Deficient Mice

#### 3.1.1. Decrease in Lipid Droplets in Subepithelial Myofibroblasts in Vitamin A-Deficient Mice

**Figure 1:**
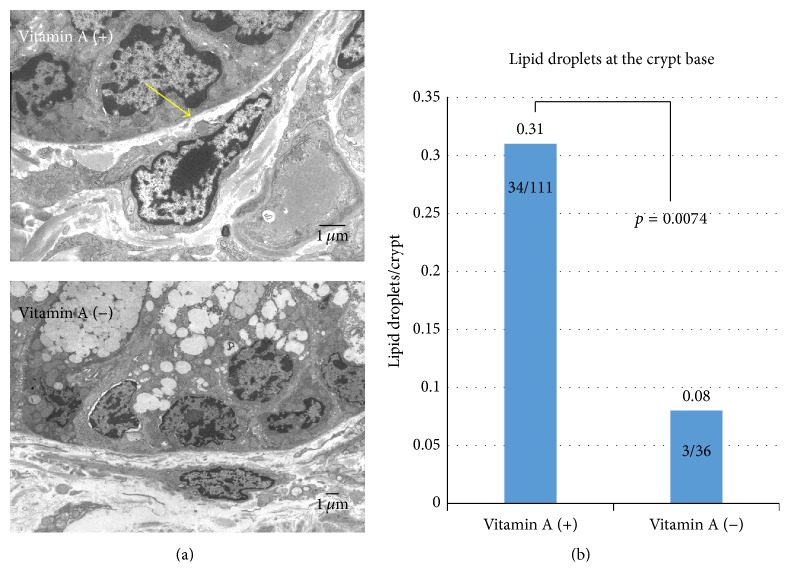
(a) Intracellular lipid droplets in mucosal subepithelial myofibroblasts of the distal segment of the colon. No lipid droplets were observed in a subepithelial myofibroblast from the colonic crypt base of a vitamin A-deficient mouse (upper) in contrast to detection of lipid droplets (arrow) in that of a vitamin A-supplemented mouse (lower) as assessed by electron microscopy. (b) Significant difference of intracellular lipid droplets in mucosal subepithelial myofibroblasts at the crypt base between two groups (Chi-squared test).

Lipid droplets identified by electron microscopy in subepithelial myofibroblasts that were located in crypt bases were significantly decreased (*p* = 0.0074) in vitamin A-deficient mice (3 lipid droplets/36 crypts, 8.3%) compared to vitamin A-supplemented mice (34/111, 30.1%) (Figure 1). Representative figures of stellate cell in the liver were shown in a vitamin A-supplemented mouse and a vitamin A-deficient mouse for the reference (see Supplementary Figure  1 in Supplementary Material available online at http://dx.doi.org/10.1155/2016/4874809).

#### 3.1.2. Increase in CD11c^+^ Dendritic Cells and Decrease in Subepithelial Myofibroblasts and Ratio of Mucosal IgA^+^/IgG^+^ Cells in Vitamin A-Deficient Mice

**Figure 2:**
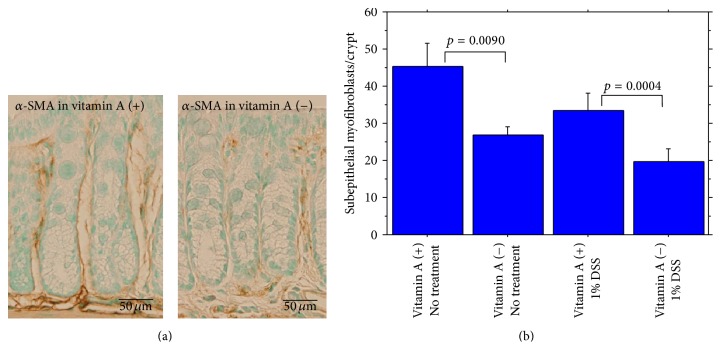
(a) Immunohistochemical *α*-smooth muscle actin (*α*-SMA)^+^ subepithelial myofibroblasts (brown) in the colonic mucosa (left, vitamin A-supplemented mouse, and right, vitamin A-deficient mouse). (b) Summary of *α*-SMA^+^ subepithelial myofibroblasts in the colonic mucosa.

**Figure 3:**
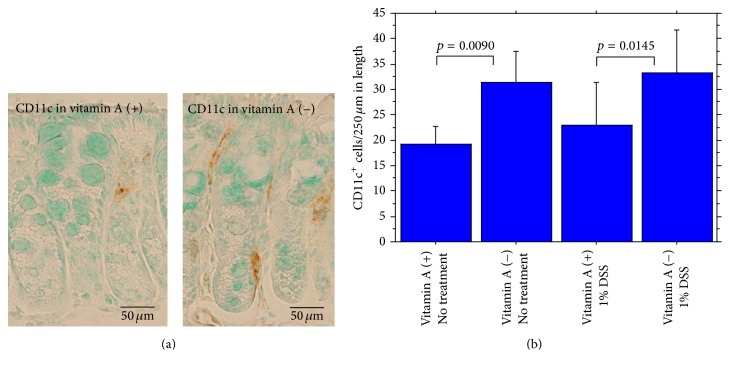
(a) Immunohistochemical CD11c^+^ dendritic cells (brown) in the colonic mucosa (left, vitamin A-supplemented mouse, and right, vitamin A-deficient mouse). (b) Summary of CD11c^+^ dendritic cells in the colonic mucosa.

**Figure 4:**
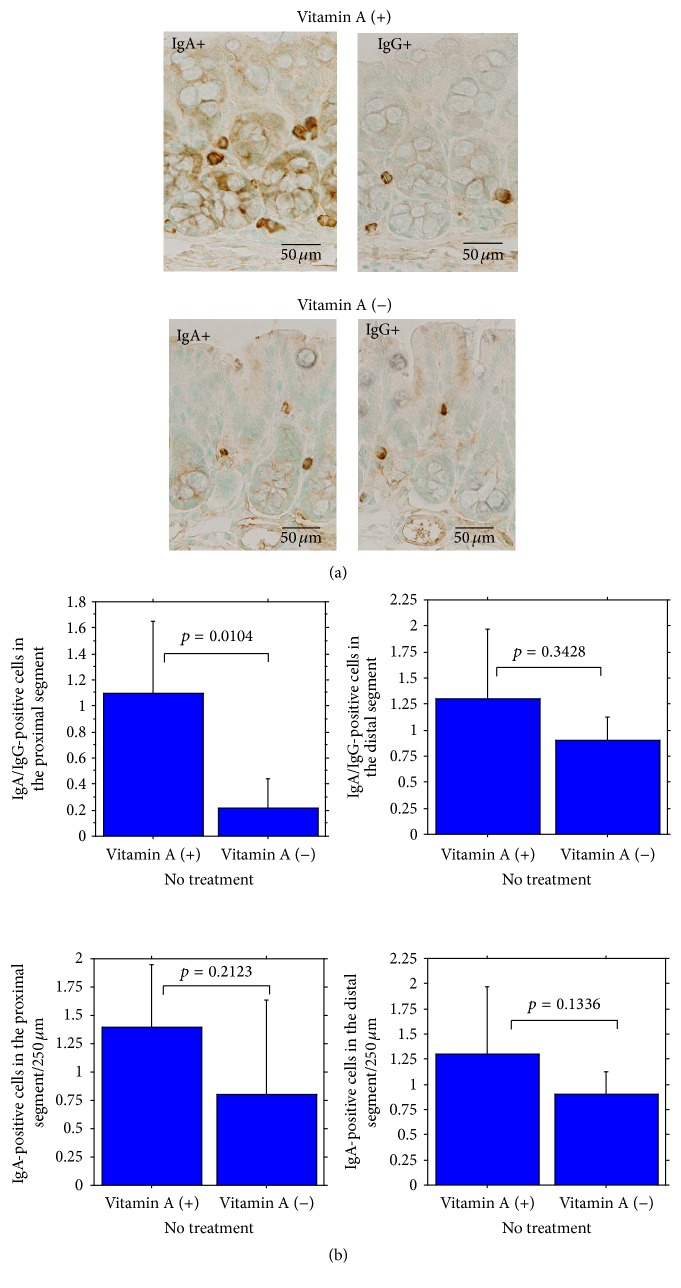
(a) Immunohistochemical IgA^+^ or IgG^+^ cells (brown) in the colonic mucosa (upper, vitamin A-supplemented mouse, and lower, vitamin A-deficient mouse). (b) Summary of the ratio of IgA^+^/IgG^+^ cells in the mucosa of the proximal (upper left) and distal segments (upper right) and IgA^+^ cells in the mucosa of the proximal (lower left) and distal (lower right) segments.

*α*-smooth muscle actin-positive (*α*-SMA^+^) subepithelial myofibroblasts were significantly decreased (Figure 2). On the contrary, CD11c-immunoreactive dendritic cells (CD11c^+^ cells/250 *μ*m of colonic length) in colonic mucosa were significantly increased in vitamin A-deficient mice compared with vitamin A-supplemented mice (Figure 3). The ratio of IgA^+^ cells (/250 *μ*m of colonic length)/IgG^+^ cells (/250 *μ*m of colonic length) in mucosa of the proximal segment was significantly lower in vitamin A-deficient mice than in vitamin A-supplemented mice but not significantly lower in mucosa of the distal segment (Figure 4). IgA^+^ cells were rather less in vitamin A-deficient mice, compared to vitamin A-supplemented mice in both proximal and distal segments, the difference being not significant.

### 3.2. Acceleration of Acute Colitis in Vitamin A-Deficient Mice

**Figure 5:**
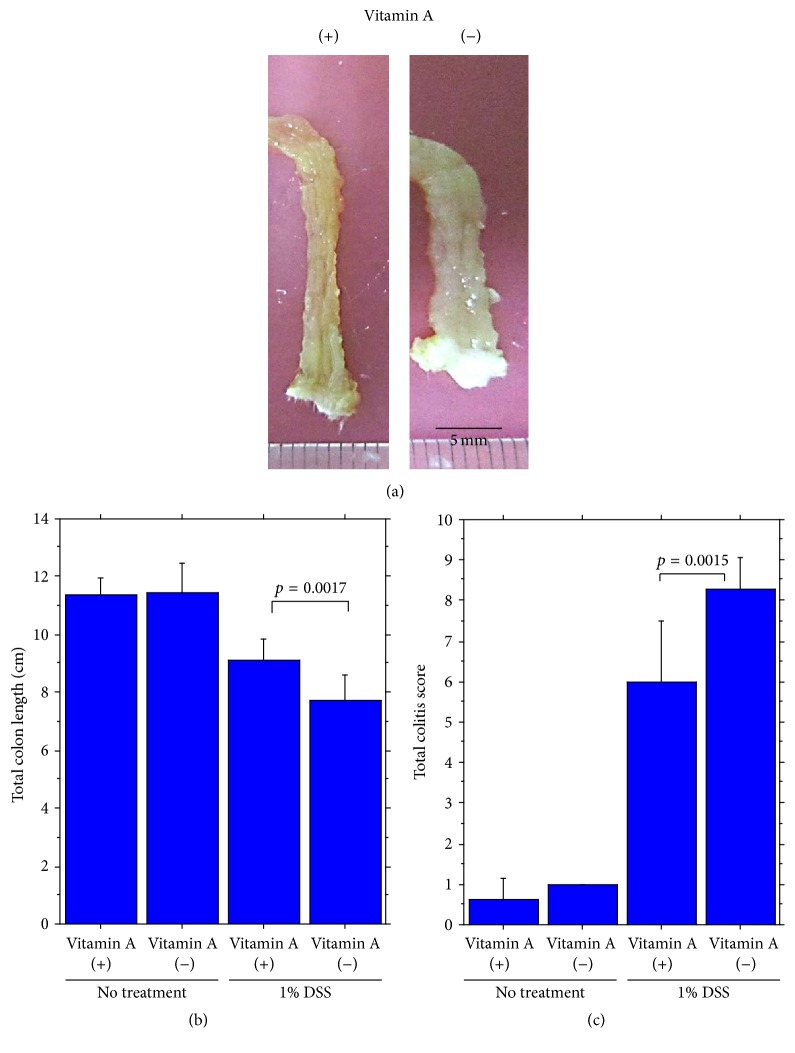
(a) Gross appearance of acute DSS colitis of the distal segment of the colon in vitamin A-supplemented mice (left) and vitamin A-deficient mice (right). In the latter there were shortening and dilatation of the colon. Colitis was induced with intake of 1% DSS for 7 days. Significant difference of (b) total colon length and (c) total colitis score between vitamin A-supplemented mice and vitamin A-deficient mice.

Acute colitis induced by oral intake of 1% DSS for 7 days was significantly more severe in vitamin A-deficient mice than in vitamin A-supplemented mice as assessed by shortening of colon length and the colitis score (Figure 5) (Supplementary Table  1a).

**Figure 6:**
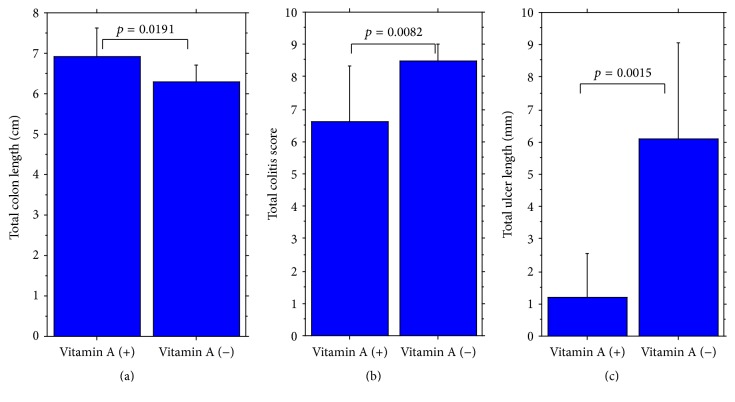
Significant difference of (a) total colon length, (b) total colitis score, and (c) total ulcer length between vitamin A-supplemented mice (left) and vitamin A-deficient mice (right). Colitis was induced with intake of 1% DSS for 12 days.

Colons of vitamin A-deficient mice were shown histologically to have undergone shortening, mild dilatation due to inflammation, gross and inflammatory granulation, and erosion (Figure 6) (Supplementary Figure  2). Differences in the degree of acute colitis induced by intake of 1% DSS for 12 days between vitamin A-deficient and vitamin A-supplemented mice were again confirmed by increased shortening of colon length, histological colitis score, and ulcer length (Figure 6) (Supplementary Table  1b).

### 3.3. Slow Recovery from Acute Colitis in Vitamin A-Deficient Mice

**Figure 7:**
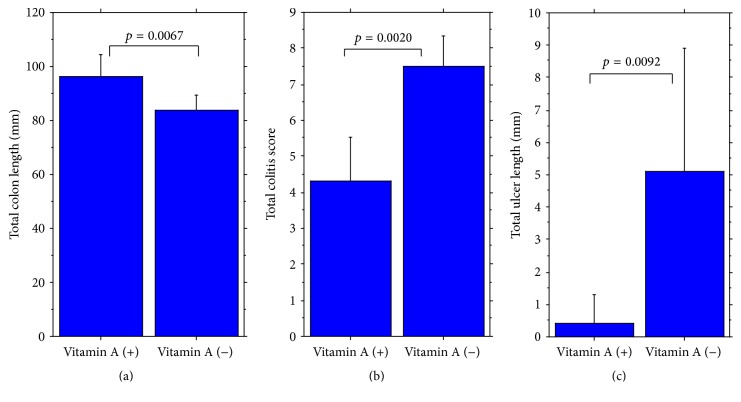
Recovery from acute colitis. Significant difference of (a) total colon length, (b) total colitis score, and (c) total ulcer length between vitamin A-supplemented mice (left) and vitamin A-deficient mice (right). Colitis was induced with intake of 1% DSS for 7 days. After that, mice were given distilled drinking water for 36 days.

Long-term observation of colitis after induction by intake of 1% DSS for 7 days revealed extensive differences between vitamin A-deficient and vitamin A-supplemented mice on experimental day 43. Vitamin A-deficient mice had much more severe colitis, including increased shortening of colon length, increased histological colitis score, and increased total ulcer length compared to vitamin A-supplemented mice (Figure 7) (Supplementary Table  1c). These findings indicate that not only the development of DSS colitis is worse with vitamin A deficiency but also recovery from DSS colitis is much slower.

### 3.4. Development of Colonic Tumors in Vitamin A-Deficient Mice

**Figure 8:**
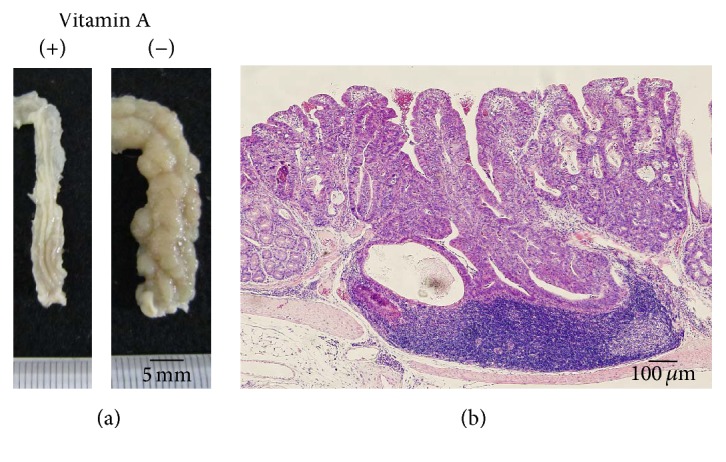
(a) Colonic neoplasia induced by a single preinjection of azoxymethane (7.4 mg/kg) 14 days prior to intake of 1% DSS for 7 subsequent days (left, no neoplasia in a vitamin A-supplemented mouse, and right, neoplasia in a vitamin A-deficient mouse). (b) Invasive adenocarcinoma.

**Figure 9:**
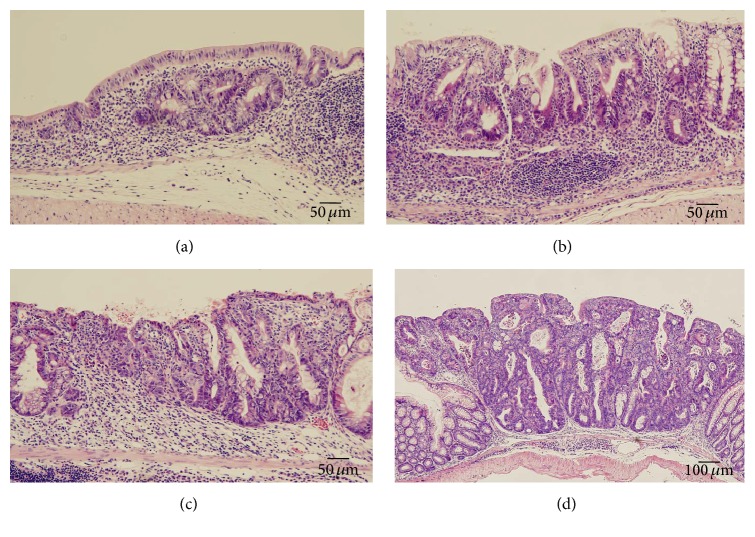
Representative dysplasia induced by a single preinjection of azoxymethane (7.4 mg/kg) 14 days prior to intake of 1% DSS for 7 subsequent days. (a) Depressed low grade dysplasia, (b) flat low grade dysplasia, (c) flat high grade dysplasia, and (d) elevated high grade dysplasia.

**Figure 10:**
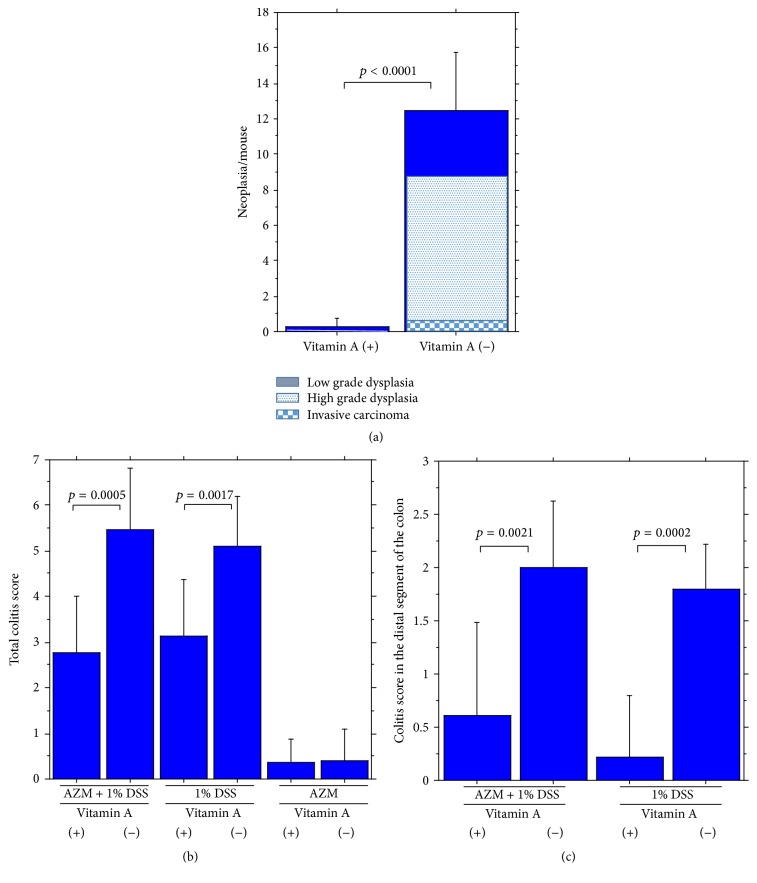
Significant difference of (a) appearing neoplasia including invasive carcinoma, high grade dysplasia, and low grade dysplasia, (b) total colitis score, and (c) colitis score of the distal segment between vitamin A-supplemented mice and vitamin A-deficient mice induced by a single preinjection of azoxymethane (7.4 mg/kg) 14 days prior to intake of 1% DSS for 7 subsequent days.

A single intraperitoneal injection of azoxymethane (7.4 mg/kg) 14 days prior to intake of 1% DSS for 7 subsequent days [7] induced multiple types of colorectal neoplasia, including low grade dysplasia, high grade dysplasia, and invasive carcinoma as well as severe colitis in vitamin A-deficient mice (Figures 8, 9, and 10). In contrast, only a few neoplastic lesions developed in vitamin A-supplemented mice in addition to mild colitis (Supplementary  Table  2).

## 4. Discussion

Our results demonstrated that vitamin A inhibits development of DSS colitis and the subsequent development of colonic neoplasia and prolonged the recovery from colitis in a mouse model of DSS colitis.

Dietary vitamin A is essential for production of the precursor of tissue retinol, which participates in immunity and cell differentiation. First, in immunity, vitamin A deficiency causes a helper T cell imbalance with excess Th1 and insufficient Th2 function and a reduction in *α*4*β*7^+^ memory/activated T cell generation [23]. In contrast, retinoic acid, a vitamin A metabolite, enhances IL-22 production by *γδ* T cells in vitro and inhibits DSS-induced colitis [24]. We demonstrated an increase in CD11c^+^ dendritic cells in the colonic mucosa of vitamin A-deficient mice in line with results of both in vivo and in vitro studies using vitamin A-deficient rats performed by Dong et al. [4]. It can be suggested that this was a compensatory increase because vitamin A deficiency induces dendritic cell malfunction in the activation of T lymphocytes [25, 26] since vitamin A supplementation was shown to induce maturation of dendritic cells [4, 25, 27]. Further, vitamin A is necessary for generation of gut-homing IgA-secreting B cells by intestinal dendritic cells [28]. In the present study, a decrease in the ratio of IgA^+^ cells/IgG^+^ cells in the mucosa of the proximal segment of colon indicates an aspect of disorganization of mucosal immunity in vitamin A deficiency [23, 28, 29]. On the other hand, present experimental results were obtained in BALB/c mice, which had higher intestinal epithelial expression of retinaldehyde dehydrogenase 1 (RALDH1), leading to an increased activity to induce IgA class switching from B cells [24]. According to this, the results might be different in another strain with low expression of RALDH1, suggesting the possibility of differences in susceptibility of ulcerative colitis in humans. This remains to be clarified.

Second, in addition, hepatic stellate cells and intestinal subepithelial myofibroblasts participate in niche function for epithelial cell stem cells and progenitor cells in the liver and intestinal crypts, respectively [17, 19, 30–34]. Hepatic stellate cells need vitamin A-rich lipids to maintain their niche function. We found a decrease in subepithelial myofibroblasts in the crypt base of vitamin A-deficient mice as well as a reduction in cytoplasmic vitamin A lipids in subepithelial myofibroblasts. The decrease in *α*-SMA^+^ subepithelial myofibroblasts may indicate an alteration of the niche function for protection of colonic mucosal stem cells or progenitor cells based on the concept that *α*-SMA^+^ subepithelial myofibroblasts correspond to colonic stellate cells [17, 19, 34]. Thus, our findings might suggest that dysfunction of dendritic cells and subepithelial myofibroblasts caused by vitamin A deficiency accelerated DSS colitis, which resulted in the development of colorectal cancer in our DSS colitis mouse model.

Third, with regard to cell differentiation, it is well known that vitamin A and its metabolite, retinoids, play an important role [6, 35]. Retinoids perform effective chemoprevention against cancers in various organs and are used for differentiation therapy against acute promyelocytic leukemia [7]. Further, it was suggested clinically and experimentally that vitamin A deficiency promotes cancer development and progression [8, 9, 36–39]. Particularly, CYP26A1, the gene encoding the cytochrome P450 enzyme specifically involved in metabolic inactivation of retinoic acid, was highly expressed in breast cancers and showed oncogenic characteristics suggesting a link between intracellular retinoic acid status and tumorigenesis [15, 40, 41]. Our results that showed severe DSS colitis, prolonged recovery of DSS colitis, and the development of colonic tumors following DSS colitis in vitamin A-deficient mice are in line with previous reports of promotion of cancer development.

Although it is suggested that dietary and nutritional factors, including vitamin A, have an intimate relationship to development or exacerbation of inflammatory bowel diseases [42, 43], there are no any definite clinical indications of vitamin A administration. Our present basic research results may help us to know the possibility that vitamin A administration might inhibit colitis and subsequent cancer development, depending on unknown phenotypes of clinical cases of ulcerative colitis, if they are present. Further studies should be conducted to find the possible underlying mechanisms on inhibition of colitis and subsequent colonic neoplasia development by vitamin A supplementation.

## 5. Conclusion

Vitamin A inhibited development of DSS colitis and colorectal cancer in the DSS colitis mouse model. Therefore, vitamin A supplementation might help to improve diarrhea and enteritis in inflammatory bowel disease and could possibly inhibit insidious colonic inflammation and cancer development. These effects of vitamin A remain to be examined in a future study.

## Supplementary Material

Supplementary FIGURE 1. Intracellular lipid droplets in a stellate cell of the liver. No lipid droplets were observed in a hepatic stellate cell of a vitamin A-deficient mouse (right) in contrast to detection of lipid droplets (arrow) in that of a vitamin A-supplemented mouse (left) as assessed by electron microscopy. Black bar indicates 1 μm in length.
Supplementary FIGURE 2. Inflammatory lesions in DSS colitis. (a) Focal inflammatory cell infiltration including polymorphonuclear leucocytes (colitis score 1). (b) Inflammatory granulation with crypt abscess (colitis score 2). (c) Inflammatory granulation with gland dropout (colitis score 2). (d) Mucosal erosion (ulceration) with inflammatory granulation (colitis score 3).
Supplementary TABLE 1. (a) Acute colitis induced with DSS drinking for 7 days (on day 8) (M±SD). (b) Acute colitis induced with DSS drinking for 12 days (on day 12) (M±SD). (c) Long term-colitis induced with DSS drinking for 7 days (on day 43) (M±SD).
Supplementary TABLE 2. Colorectal neoplasia and colitis induced with a combination of azoxymetane preinjection and DSS drinking for 7 days (on day 28) (M±SD).

## Figures and Tables

**Table 1:** Antibodies and immunohistochemical examination.

Antibody	Clone	Source	Fixation	Dilution	Antigen retrieval	2nd step and colorization	Interpretation
CD11c	Hamster anti-mouse CD11c	BD Pharmingen 550283	2% paraformaldehyde, frozen sections	×10	Not applied	Streptavidin Biotin/HRP kit (Jackson ImmunoResearch, West Grove, PA)	Dendritic cells

*α*-SMA	Monoclonal mouse anti-*α*-SMA	DAKO M851 (DakoCytomation, Glostrup, Denmark)	10% buffered formalin, paraffin sections	×1000	Not applied	Histofine mouse staining kit (Nichirei, Tokyo, Japan)	Smooth muscle actin

IgM	Goat polyclonal	Abcam ab98673 (Abcam, Cambridge, MA)	10% buffered formalin, paraffin sections	×500	Microwave 5 min × 3 times	Streptavidin Biotin/HRP kit (Jackson ImmunoResearch)	IgM^+^ lymphocytes

IgG	Goat polyclonal	Abcam ab98802 (Abcam, Cambridge, MA)	10% buffered formalin, paraffin sections	×500	Microwave 5 min × 3 times	Streptavidin Biotin/HRP kit (Jackson ImmunoResearch)	IgG^+^ lymphocytes

IgA	Goat polyclonal	Abcam ab97233 (Abcam, Cambridge, MA)	10% buffered formalin, paraffin sections	×500	Microwave 5 min × 3 times	Streptavidin Biotin/HRP kit (Jackson ImmunoResearch)	IgA^+^ lymphocytes

*α*-SMA: *α*-smooth muscle antigen.
