# Supplementary material for: Vitamin A Inhibits Development of Dextran Sulfate Sodium-Induced Colitis and Colon Cancer in a Mouse Model
Source: Biomed Res Int. 2016 May 19;2016:4874809. doi: 10.1155/2016/4874809 (PMC4889797; doi:10.1155/2016/4874809)
Supplement: Supplementary file 1 — Supplementary FIGURE 1. Intracellular lipid droplets in a stellate cell of the liver. No lipid droplets were observed in a hepatic stellate cell of a vitamin A-deficient mouse (right) in contrast to detection of lipid droplets (arrow) in that of a vitamin A-supplemented mouse (left) as assessed by electron microscopy. Black bar indicates 1 μm in length. Supplementary FIGURE 2. Inflammatory lesions in DSS colitis. (a) Focal inflammatory cell infiltration including polymorphonuclear leucocytes (colitis score 1). (b) Inflammatory granulation with crypt abscess (colitis score 2). (c) Inflammatory granulation with gland dropout (colitis score 2). (d) Mucosal erosion (ulceration) with inflammatory granulation (colitis score 3). Supplementary TABLE 1. (a) Acute colitis induced with DSS drinking for 7 days (on day 8) (M±SD). (b) Acute colitis induced with DSS drinking for 12 days (on day 12) (M±SD). (c) Long term-colitis induced with DSS drinking for 7 days (on day 43) (M±SD). Supplementary TABLE 2. Colorectal neoplasia and colitis induced with a combination of azoxymetane preinjection and DSS drinking for 7 days (on day 28) (M±SD). [file 4874809.f1.docx]

**SUPPLEMENTARY TABLES**

**SUPPLEMENTARY TABLE 1**

**SUPPLEMENTARY TABLE 2**

**SUPPLEMENTARY FIGURE 1**

**SUPPLEMENTARY FIGURE 2**

**SUPPLEMENTARY FIGURE Legends**

**SUPPLEMENTARY FIGURE 1:** Intracellular lipid droplets in a stellate cell of the liver. No lipid droplets were observed in a hepatic stellate cell from the liver of a vitamin A-deficient mouse (right) in contrast to detection of lipid droplets (arrow) in that of a vitamin A-supplemented mouse (left).

**SUPPLEMENTARY FIGURE 2:** Inflammatory lesions in DSS colitis. **(a)** Focal inflammatory cell infiltration including polymorphonuclear leucocytes (colitis score 1). **(b)** Inflammatory granulation with crypt abscess (colitis score 2). **(c)** Inflammatory granulation with gland dropout (colitis score 2). **(d)** Mucosal erosion (ulceration) with inflammatory granulation (colitis score 3).
